# Analysis of ABCB1 Gene Polymorphisms and Their Impact on Tacrolimus Blood Levels in Kidney Transplant Recipients

**DOI:** 10.3390/ijms252010999

**Published:** 2024-10-12

**Authors:** Corina Andreea Rotarescu, Ion Maruntelu, Ion Rotarescu, Alexandra-Elena Constantinescu, Ileana Constantinescu

**Affiliations:** 1 Department of Immunology and Transplant Immunology, “Carol Davila” University of Medicine and Pharmacy, 37 Dionisie Lupu Street, 020021 Bucharest, Romania; corina.rotarescu@drd.umfcd.ro (C.A.R.); alexandra-elena.constantinescu0720@stud.umfcd.ro (A.-E.C.); ileana.constantinescu@imunogenetica.ro (I.C.); 2 Department of Immunogenetics and Virology, Fundeni Clinical Institute, 258 Fundeni Avenue, 022328 Bucharest, Romania; 3 Department of Cardiovascular Surgery, “Prof. Dr. C. C. Iliescu” Emergency Institute for Cardiovascular Diseases, 258 Fundeni Avenue, 022328 Bucharest, Romania; rtrsc_i@yahoo.com; 4 Academy of Romanian Scientists (AOSR), 3 Ilfov Street, 030167 Bucharest, Romania

**Keywords:** kidney transplant, ABCB1, tacrolimus, haplotype, diplotype

## Abstract

Tacrolimus (Tc) is an immunosuppressant used in transplant patients, but its therapeutic range is narrow, making precise dosing essential. This study investigates the association of three single nucleotide polymorphisms (SNPs) (ABCB1 3435C>T, 1236C>T, 2677G>T/A) with Tc levels over time to gain better insights into their role in personalized medicine. We conducted the study over four distinct periods: 1–14 days, 15–30 days, 31–60 days, and beyond 60 days post-transplantation. The analysis included allele, genotype, haplotype, and diplotype frequencies of the three SNPs concerning Tc blood levels. Statistical significance was determined, and false discovery rate (*P*_FDR_) correction was applied where appropriate. Significant associations were found between the C (ABCB1 C1236T), A alleles (ABCB1 G2677T/A), the CAC haplotype and lower Tc levels. The CAC-TGT and TGT-TGT diplotypes significantly influence how patients metabolize the drug. The TGT haplotype and the AA genotype (ABCB1 G2677T/A) were associated with higher Tc levels, suggesting a long-term genetic influence. Genetic factors, specifically certain SNPs and diplotypes, significantly impact Tc blood levels, with their influence varying over time.

## 1. Introduction

Chronic kidney disease is a progressive disorder characterized by a gradual decline in kidney function over months or years. Worldwide, an estimated 850 million people suffer from kidney disease, with the majority living in lower-middle-income nations, where they lack access to therapy, prevention and diagnosis [1]. End-stage renal disease is the most severe chronic kidney disease [2]. A glomerular filtration rate of fewer than 15 milliliters per minute is the hallmark of this disease, characterized by severe and irreversible kidney damage [3,4]. Treatments for end-stage renal disease primarily consist of renal replacement therapy or kidney transplants. Kidney transplantation is an indication of the implementation of immunosuppressive therapy. The most common immunosuppressive treatment involves Anti-thymocyte globuline or Basiliximab for initial induction. Maintenance includes Prednisone, Mycophenolate mofetil, Tacrolimus (Tc), or Cyclosporine.

Tc is one of the most studied ABCB1 substrates because it is frequently used to prevent transplant rejection [5]. Bioavailability changes significantly impact Tc pharmacokinetics. The drug’s oral bioavailability is poor and varies considerably between people, with an average of about 25% [6,7,8].

The ABCB1 gene, on chromosome 7 at position 7q21.12 is highly polymorphic and encodes the P-glycoprotein (a transmembrane protein) [9]. The protein’s molecular weight is 170 kDa [10], and it is present in the epithelia of numerous tissues, including the intestines, liver, kidneys, blood–brain barrier, testes, placenta, and lung [11,12,13]. ABCB1 gene polymorphisms are also linked to different pathologies, not only to drug metabolism. For instance, ABCB1 intron 3 C-rs3789243-T is associated with colorectal cancer (CRC) risk, as found in a prospective population-based study [14]. Similarly, a meta-analysis of case–control studies found an increased risk of CRC among carriers of the combined wild-type C3435T and G2677T/A alleles in Caucasians [15].

The ABCB1 gene polymorphisms can alter the structure and function of the P-glycoprotein, a key player in drug distribution and efficacy. These variations can affect the P-glycoprotein’s ability to expel drugs from cells, thereby influencing the distribution and efficacy of Tc in kidney transplantation.

Three polymorphic variants, ABCB1 3435C>T (exon 26), ABCB1 1236C>T (exon 12) and ABCB1 2677G>T/A (exon 21), have been extensively analyzed and demonstrated to hold functional significance in the context of kidney transplantation.

Our study aims to present findings from a retrospective single-center study on the impact of ABCB1 gene polymorphisms on Tc dosage in Romanian kidney transplant recipients. The goal is to contribute to optimizing Tc administration for kidney transplant recipients. Based on the findings of this study, individualized Tc administration may minimize the time required for optimal Tc levels with minimal adverse effects in clinical practice and reduce the risk of rejection in Romanian kidney transplant recipients.

## 2. Results

### 2.1. Demographic Data and Gene Frequencies

Table 1 presents a detailed summary of 162 patients, including their medical history, demographics, and specific treatments. The average age of the patients is 40.68, with 65.4% being men. A significant finding is that over 40% of the patients have an unknown cause of their chronic kidney disease, highlighting the need for further investigation in this area. Regarding the donor type, 74.7% received kidneys from living donors, while 25.3% received kidneys from cadaveric donors.

Table 2 presents the frequency distributions of genotypes for genetic variants in the ABCB1 gene. The allelic distribution of the G2677T/A variant was 70.4% for G, 29.6% for A, and 0% for T. The genotype frequencies for this variant were as follows: 28.5% (46/162) for homozygous wild-type, 41.9% (68/162) for heterozygous, and 29.6% (48/162) for homozygous mutant. Notably, no patients exhibited a GT/TA/TT genotype for this locus, highlighting the rarity of this genotype in the population under study. The C and T alleles were 75.3% and 24.7% for the C3435T SNP and 70.4% and 29.6% for the C1236T SNP, respectively. Regarding the genotypes ABCB1 3435C>T and ABCB1 1236C>T, the homozygous mutant was observed in 24.7% and 29.6% of patients. Notably, the genotype distribution adhered to the Hardy–Weinberg equilibrium, validating our research methodology and the reliability of our results.

### 2.2. ABCB1 SNP Alleles and Genotypes Analysis

The association between each SNP of the ABCB1 alleles and genotype and the Tc C0 was analyzed.

**Figure 1:**
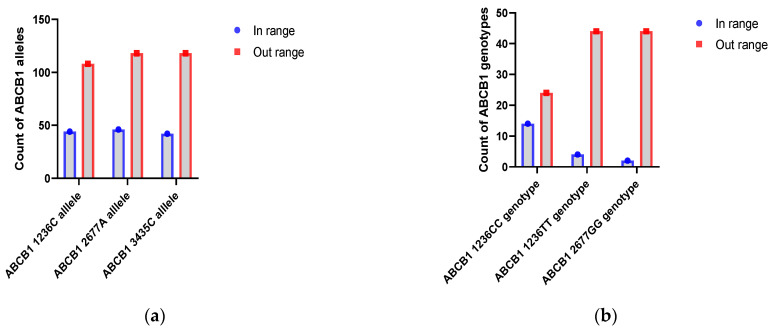
Count of ABCB1 alleles and genotypes with Tc levels outside the normal range in the first two weeks: (**a**) Distribution of ABCB 1 with abnormal Tc levels. (**b**) Distribution of ABCB1 genotypes with abnormal Tc levels.

During the 1–14 day period, allele analysis revealed that the C and A alleles of ABCB1 C1236T and ABCB1 G2677A were significantly more frequent in the group with Tc levels outside the normal range, with *p*-values of 0.0009 (OR = 0.39; 95% C.I. = 0.22–0.69) and 0.0015 (OR = 0.41; 95% C.I. = 0.23–0.72), respectively. The C allele of ABCB1 C3435T also showed a higher frequency in the outside range group (*p* = 0.0215; OR = 0.53; 95% C.I. = 0.31–0.91) (Figure 1a)

Patients homozygous on ABCB1 C1236T and GG from locus ABCB1 G2677T/A usually were outside of the normal range compared to heterozygotes status (ABCB1 1236CC with *p* = 0.0061; *P*_FDR_ = 0.0182; 95% C.I. = 0.01–0.04; ABCB1 1236TT with *p* = 0.0102; *P*_FDR_ = 0.0154; 95% C.I. = 0.01–0.09; ABCB1 2677GG with *p* = 0.0010; *P*_FDR_ = 0.0031; 95% C.I. = 0–0.01).

During the first two weeks after kidney transplantation, ABCB1 1236C, ABCB1 2677A and ABCB1 3435C alleles have been associated with low blood levels of Tc. This also applies to the CC and TT genotypes from the ABCB1 C1236T locus and the GG genotype from the ABCB1 G2677T/A locus (Figure 1b).

Beyond 60 days, significant associations re-emerged. The C allele of ABCB1 C1236T and the A allele of ABCB1 G2677A were associated with the standard range group, with *p*-values of 0.0317 and 0.0106, respectively. Genotype analysis revealed that the AA genotype of ABCB1 G2677A was significantly associated with Tc levels within the normal range (*p* = 0.0111; *P*_FDR_ = 0.0334; 95% C.I. = 0.01–0.08).

**Figure 2:**
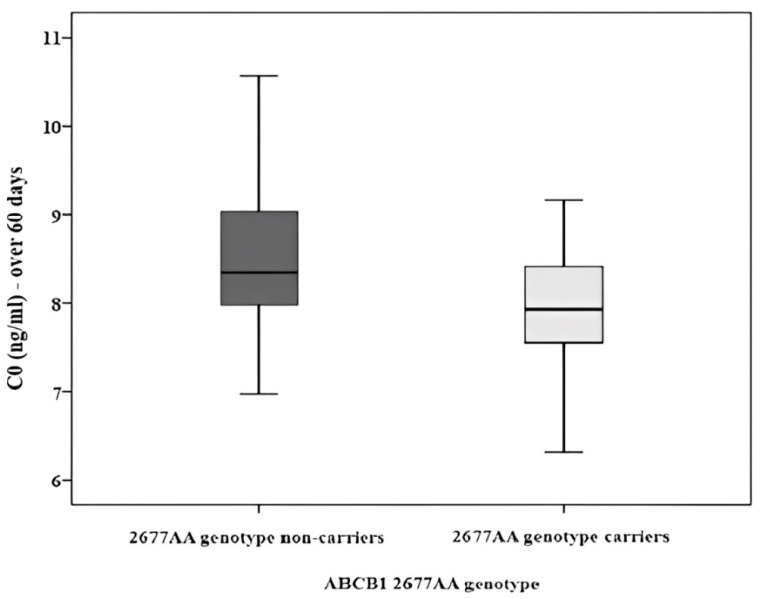
Blood concentration of Tc over 60 days for ABCB1 2677 AA genotype.

Patients carrying the AA genotype from the ABCB1 2677 group tend to have lower concentrations than those without the AA genotype, as indicated by the lower mean rank (29.67 vs. 8.33) and *p*-value = 0.005 (Figure 2). However, no other significant differences in Tc C0 were observed among ABCB1 1236C>T and 3435C>T genotype groups.

### 2.3. ABCB1 Haplotypes and Diplotypes Analysis

Patients who were homozygous for three variants or heterozygous for only one variant had their haplotypes (composed of alleles belonging to ABCB1 C1236T, ABCB1 G2677T/A and ABCB1 C3435T loci) unambiguously assigned, as reported by Lee et al. [16]. We used the Haploview software for the remaining patients to assign the haplotypes using the estimation-maximization algorithm.

**Figure 3:**
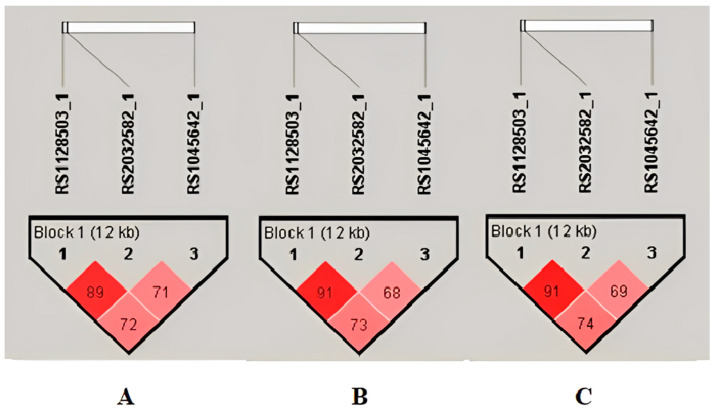
Linkage disequilibrium plots and haplotype analysis during periods of 1–14 days (**A**) and 31–60 days (**B**), respectively, over 60 days (**C**) with Tc levels in the outside range. For D’ < 1, LOD ≥ 2, use a shade of pink; for D’ = 1, LOD ≥ 2, use bright red.

A single haplotype block (formed by the G2677T/A, C3435T and C1236T) was identified in the Romanian group chromosome 7 region by the linkage disequilibrium (LD) analysis (Figure 3).

Haplotype analysis indicated that the CAC haplotype was more prevalent in the abnormal range group (*p* = 0.0006, *P*_FDR_ = 0.0051) in the first two weeks.

We have observed no significant associations in allele or haplotype frequencies with the status range of Tc levels during the third and fourth weeks, following a kidney transplant. Genotype and diplotype analyses also showed no notable differences, except for the CAC-TGT diplotype, which had a marginal association with Tc levels. A marginal association indicates a weak but present relationship, with a *p*-value of 0.0229 and *P*_FDR_ = 0.3219. In other words, while the relationship is not strong enough to be considered significant, it is still worth noting as it may have some influence.

During the 31–60 day period, the analysis showed no significant differences in allele frequencies. However, the TGC haplotype was significantly more frequent in the standard range group (*p* = 0.0048, *P*_FDR_ = 0.0385). Diplotype analysis supported this finding, with the TGC-TGC diplotype being exclusive to the normal range group.

Beyond 60 days, the TGT haplotype was significantly more prevalent in the group with Tc levels in the normal range (*p* = 0.0093, *P*_FDR_ = 0.0467).

**Figure 4:**
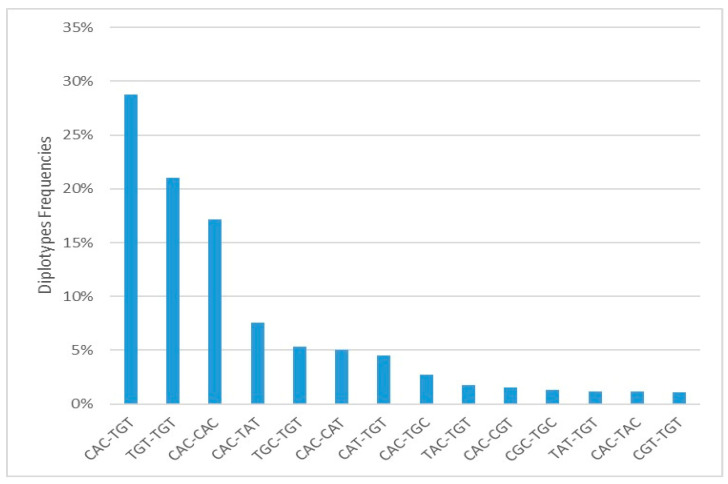
Diplotype frequency of combined ABCB1 1236C>T, 2677G>T/A and 3435C>T.

Out of 162 patients, 64.20% had heterozygous diplotypes (Figure 4). All the identified ABCB1 diplotypes were used to compare the Tc C0 levels at different periods since the transplant procedures.

Diplotype analysis found the TGT-TGT (*p* = 0.0018, *P*_FDR_ = 0.0213) and CAC-TGT (*p* = 0.0070, *P*_FDR_ = 0.0421) diplotypes more frequent in the abnormal range group, suggesting a potential impact on Tc levels.

### 2.4. Analysis of Tc Variability (C0/Dose Ratio) over Time through Different Mixed-Effects Models

A mixed-effects logistic regression model was employed to account for patient variability. This model considers the uniqueness of each patient’s baseline Tc C0/dose. Including patient-specific random effects in the model improved the AUC (Area Under the Curve) from 0.615 to 0.829, indicating a significantly better ability to predict which patients will have high Tc concentrations. This suggests that accounting for patient-specific variability is crucial in predicting Tc levels, emphasizing the need for individualized therapeutic monitoring.

Only during the 1 to 14 days were CAC-TGT and TGT-TGT diplotypes significantly associated with lower Tc blood levels. This suggests that patients with these specific genetic variations may metabolize Tc more efficiently, leading to lower drug concentrations.

Patients with the CAC-TGT diplotype had a 99% reduced likelihood of exhibiting high Tc concentrations, and those with the TGT-TGT diplotype had a 72% reduced likelihood. These findings were robust even after correcting for multiple comparisons using the False Discovery Rate (P_FDR_) method, confirming the genetic variations as essential determinants of Tc pharmacokinetics.

Age showed a weak trend toward influencing Tc levels (*p* = 0.07), with older patients slightly more likely to have higher concentrations. eGFR, an essential marker of kidney function, was not significantly associated with Tc levels in this model (*p* = 0.11), suggesting that other factors, such as genetic variations, may play a more direct role in influencing drug concentration.

After 15 days of kidney transplantation, age and eGFR were significantly associated with Tc variability. Patients with higher eGFR, indicating better kidney function, were less likely to have elevated Tc levels (OR: 0.32, *p* < 0.001), whereas older patients had increased odds of higher Tc C0 (OR: 0.31, *p* = 0.005). Dose per weight was a critical factor, as patients receiving higher Tc C0/dose/bodyweight were much more likely to have higher blood levels (OR: 1.02, *p* < 0.001).

## 3. Discussion

The impact of ABCB1 polymorphisms on drug response varies significantly across ethnic populations, influencing drug bioavailability and efficacy. Understanding these genetic differences is essential for developing personalized medicine strategies to optimize therapy based on an individual’s genotype.

Our study uniquely examines the relationship between alleles, genotypes, haplotypes, diplotypes, and clinical factors on Tc levels at different time intervals (1–14 days, 15–30 days, 31–60 days, and over 60 days) in Romanian kidney transplant recipients. The term ‘haplotype’ refers to a combination of alleles on specific chromosomes within a population [17], while ‘diplotype’ describes the combination of genotypes formed by homologous chromosome pairs [17].

In this study, the frequency of the variant C allele for ABCB1 C1236T and ABCB1 C3435T SNPs was 70.4% and 75.3%, differing from the Turkish population [18]. According to dbSNP [19], the ABCB1 1236C allele is the minor variant in Asians but the major variant in other populations, highlighting ethnic variability. The ABCB1 3435C allele also varies widely in frequency, being more common in some populations and less common in others [20,21,22]. The role of the ABCB1 1236C, ABCB1 2677A and ABCB1 3435C, along with the CAC haplotype, as potential risk factors for abnormal Tc levels aligns with findings from previous studies. In our research, beyond 60 days, kidney transplant recipients with the AA genotype in the ABCB1 2677 group tended to have a normal Tc concentration compared to patients without the AA genotype.

ABCB1 2677GG, homozygous ABCB1 C1236T is associated with abnormal Tc blood levels in the first two weeks.

In a study of Korean kidney transplant patients, carriers of the ABCB1 2677 GG genotype exhibited lower Tc dose-adjusted trough levels on day three after transplantation than non-GG carriers [23]. Fredericks et al. [24] examined the Tc dose requirements of 206 stable renal transplant patients. They discovered that ABCB1 G2677G and ABCB1 C3435C genotype patients had lower dose-normalized blood Tc concentrations than ABCB1 T2677T and ABCB1 T3435T patients [24]. Additionally, a research team led by Karim Akbas [25], conducted a study on the influence of ABCB1 polymorphism on Tc pharmacokinetics in Turkish individuals who had received a kidney transplant. The study findings showed that among the participants, 30.4% had the ABCB1 C3435C genotype, 47.8% had the ABCB1 C3435T genotype, and 21.7% had the ABCB1 T3435T genotype [25]. During the first six months after transplantation, patients with the 3435 TT genotype required significantly lower daily Tc [25]. However, in the following six months, individuals with the 3435 CC genotype had significantly lower trough Tc concentrations adjusted for dose than those with the 3435 TT and CT genotypes [25].

Nonetheless, a study by Provenzani et al. found that kidney transplant recipients with the wild-type genotype ABCB1 2677GG required significant doses of Tc [26]. Most scientists explored gene variants in ABCB1 and found no statistically significant association with dose-adjusted Tc trough levels [27,28]. This suggests that in the population studied, the ABCB1 genotype did not exert a notable impact on the pharmacokinetics of the medications transported by this gene. This could be attributed to several factors. These include the influence of other genes, environmental factors, and the limitations of the study design. Additional investigations are required to validate these findings and understand the complex interplay between genetics and transport.

Our findings indicate that the CAC haplotype (1–14 days) and TGC haplotype (31–60 days) are associated with abnormal Tc levels. The genetic influence on Tc metabolism diminishes over time, with non-genetic factors potentially playing a more significant role. As outlined in our hospital protocol, patients with the CAC haplotype required lower Tc doses during the first two weeks post-transplant to reach therapeutic levels.

The high frequency of CAC and TGT haplotypes may suggest evolutionary stability or a selective advantage in this population. Identifying CAC-TGT and TGT-TGT diplotypes as predictors of lower tacrolimus blood levels could aid in tailoring tacrolimus dosing. Due to enhanced drug metabolism, patients with these diplotypes may need higher doses to achieve therapeutic Tc concentrations. Genetic testing for ABCB1 polymorphisms could be integrated into routine post-transplant care, enabling more personalized dosing strategies.

The applied mixed-effects model highlights the importance of personalized medicine in managing kidney transplant recipients. While genetic factors significantly influence Tc metabolism, other factors, such as age, clinical status, and lifestyle, must be considered. Incorporating patient-specific variability into predictive models could improve dosing accuracy, preventing under- or over-dosing, which are associated with risks of transplant rejection or toxicity. The model also supports frequent therapeutic drug monitoring to ensure Tc levels remain within the target range.

Age significantly impacted Tc levels after 15 days, with older patients tending to have higher concentrations, likely due to reduced hepatic and renal clearance. Interestingly, despite being a key indicator of kidney function, eGFR did not significantly predict Tc concentration, possibly due to the dominant influence of genetic factors in this context.

Our findings suggest that genetic testing could inform personalized treatment protocols, especially regarding Tc dosing. Early dose adjustments based on genetic profiles may reduce the risk of acute rejection and tacrolimus-related nephrotoxicity. Routine monitoring should also account for individual variability, particularly in the early post-transplant months when Tc levels are more variable.

The small sample size for certain genetic variants can reduce the statistical power of the study, making it difficult to draw definitive conclusions about the impact of these genetic variants on Tacrolimus concentrations and dosing. Without randomization and a control group, it is challenging to establish causality. The observed effects might be influenced by confounding factors such as donor age and type that were not controlled for in the study design. Conducting the study at a single center limits the generalizability of our findings. The results may not apply to broader populations with different demographic or clinical characteristics. Retrospective studies are prone to various biases, which can affect the validity of the findings.

## 4. Materials and Methods

**Figure 5:**
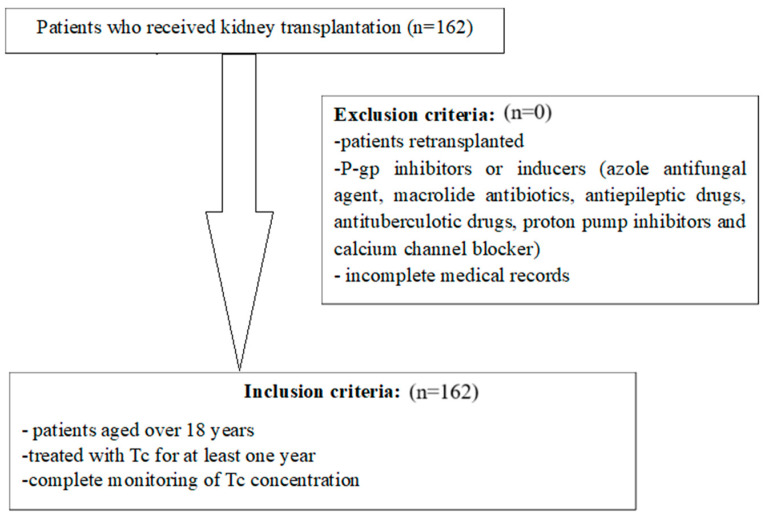
Design of the current study.

This study included 162 kidney transplant recipients treated with Tc (*Prograf or *Advagraf) for at least one year between January 2019 and May 2024 at Fundeni Clinical Institute, Bucharest, Romania (see Figure 5). Tc was administered orally once or twice daily with an initial dose of 0.15–0.20 mg/kg/day and then adjusted doses to achieve target concentrations: 12–15 ng/mL in the first 14 days, 10–12 ng/mL during 15–30 days, 8–10 ng/mL during 31–60 days, and 6–8 ng/mL after 60 days. All patients had normal liver function throughout the follow-up period. Therefore, we analyzed only: age, sex, renal function by eGFR, dose, concentration, and ABCB1 genes. The data on demographics, laboratory tests, underlying medical conditions, and medication during the perioperative period for this study were collected retrospectively from the electronic medical system (Hipocrate system). The study was conducted following the Declaration of Helsinki (2013) and was approved by the Commission of Ethics (24433/23.05.2024).

Tc concentrations (C0, ng/mL) were monitored biweekly for the first month following kidney transplantation and then monthly until the end of the first year. C0 refers to the Tc trough concentration, measured just before the next dose is administered (pre-dose). This measurement reflects the lowest concentration of the drug in the bloodstream, providing an indicator of drug exposure, and is commonly used to guide dosing adjustments in clinical practice. The chemiluminescent microparticle immunoassay method was used to measure Tc C0 in whole blood (on Architech System^®^ i2000 from Abbott Diagnostic, Lake Forest, IL, USA). In our laboratory, the measurement range (2–30 ng/mL) and detection limit (1 ng/mL) align with the manufacturer’s specifications [29].

DNA was isolated from 200 µL of whole blood using the QIAmp DNA Blood Mini Kit (Qiagen, Germany). The DNA was analyzed for the genes ABCB1 1236C>T (rs1128503), 2677G>T/A (rs2032582) and 3435C>T (rs1045642) using TaqMan^®^ Drug Metabolism Genotyping Assays. The TaqMan^®^ Drug Metabolism Genotyping Assays were chosen for their high specificity, sensitivity, and reliability, making them well-suited for analyzing the genetic influence on drug metabolism. The analysis was performed on an Applied Biosystems 7300 Real-Time PCR System.

Descriptive statistics were used to represent the demographic data and gene frequencies. Before using parametric or nonparametric tests, we checked for the normal distribution of our data using tests for normality such as the Shapiro–Wilk or Kolmogorov–Smirnov Test. Variables like C0/dose and estimated glomerular filtration rate (eGFR) were not normally distributed, so we log-transformed them for analysis.

We used T-student or Chi-square tests to study SNP alleles and Tc levels’ in/out range status as needed. Nonparametric tests were used to examine the impact of genotype, haplotype, and diplotype groups on Tc C0/dose. The groups were compared using the Mann–Whitney U test, and we utilized the SPSS software (v.20, IBM SPSS Statistics for Windows, Armonk, NY, USA) for analysis. GraphPad Prism version 9.3 for Windows (GraphPad Software, San Diego, CA, USA) was used for plotting graphs.

Hardy–Weinberg equilibrium, linkage disequilibrium and haplotype frequency analyses were determined using the Haploview 4.2 program (Broad Institute of Harvard and MIT, Cambridge, MA, USA).

A linear mixed-effects model was used to assess the impact of various covariates on Tc C0/dose over different time intervals (1–14 days, 15–30 days, 31–60 days, and over 61 days post-transplant). The model included age, gender, weight, time since transplant, and eGFR. The R package version 4.4.1 was used to analyze the mixed-effect model. The false discovery rate (P_FDR_) method was used to reduce the likelihood of false positives in multiple comparisons [30]. The association was considered statistically significant if the *P*_FDR_ was less than 0.05.

## 5. Conclusions

The study’s results underscore the importance of considering the temporal dynamics of genetic influence on Tc metabolism. The fluctuating significance of genetic markers over time suggests that personalized Tc therapy could benefit from a longitudinal approach, where genetic testing is not just a one-time assessment but is integrated into ongoing treatment evaluations. This approach can provide a more comprehensive understanding of the genetic influence on Tc levels, leading to more effective and personalized treatment strategies.

## Figures and Tables

**Table 1:** Characteristics of the patients.

**Number of Patients (n)**	**162**			
**Age (mean ± standard deviation)**	40.68 ± 11.26			
**Gender (n)**				
Male	106 (65.4%)			
Female	56 (34.6%)			
**Bodyweight (kg)** **(mean ± standard deviation)**	69.69 ± 14.85			
**Blood groups (n)**				
A	74 (45.7%)			
B	32 (19.7%)			
AB	18 (11.1%)			
O	38 (23.5%)			
**Type of donor (n)**				
Living donor	121 (74.7%)			
Cadaveric donor	41 (25.3%)			
**Kidney disease causes (n)**				
Unknown etiology	67 (41.4%)			
IgA nephropathy	29 (17.9%)			
Autosomal dominant polycystic kidney	14 (8.6%)			
Glomerulonephritis	12 (7.4%)			
Tubulointerstitial disease	11 (6.8%)			
Alport syndrome	10 (6.2%)			
Diabetic nephropathy	8 (4.9%)			
Hypertensive nephropathy	3 (1.9%)			
Others (LES, ANCA positive vasculitis, Fabry disease, Goodpasture syndrome)	8 (4.9%)			
**Induction therapy (n)**				
Anti-thymocyte globuline	2 (1.2%)			
Basiliximab	160 (98.8%)			
**Characteristics over time** **(mean ± standard deviation)**	**1–14 days**	**15–30 days**	**31–60 days**	**over 60 days**
**Tc C0 (ng/mL)**	12.18 ± 5.42	13.55 ± 4.42	11.53 ± 3.65	8.15 ± 2.91
**Tc dose (mg per day)**	12.92 ± 4.19	12.43 ± 4.89	8.89 ± 4.38	4.99 ± 2.56
**Tc C0/dose (ng/mL/mg per day)**	1.11 ± 1.20	1.29 ± 0.83	1.59 ± 0.87	1.98 ± 1.12
**Tc C0/dose/Bodyweight (ng/mL/mg/kg per day)**	0.016 ± 0.155	0.019 ± 0.151	0.024 ± 0.015	0.029 ± 0.016
**eGFR (mL/min/1.73 m^2^)**	37.87 ± 22.23	37.53 ± 21.49	43.98 ± 14.76	47.29 ± 15.81

**Table 2:** Genotype and allele frequencies of the patients.

Genotype	Total (n = 162)	Allele	Total (n = 324)
**ABCB1 3435C>T (rs1045642)** CC CT TT	38 (23.4%) 84 (51.9%) 40 (24.7%)	C T	244 (75.3%) 80 (24.7%)
**ABCB1 1236C>T (rs1128503)** CC CT TT	38 (23.5%) 76 (46.9%) 48 (29.6%)	C T	228 (70.4%) 96 (29.6%)
**ABCB1 2677G>T/A (rs2032582)** GG GA AA GT/TA/TT	46 (28.5%) 68 (41.9%) 48 (29.6%) 0 (0%)	G A T	228 (70.4%) 96 (29.6%) 0 (0%)

## Data Availability

The dataset is available on request from the authors.
